# Mr. Hume, on Modern Pharmacopoeias

**Published:** 1804-07-01

**Authors:** J. Hume

**Affiliations:** Long Acre


### *To the Editors of the Medical and Phyſical Journal.*

GENTLEMEN,

I Again lay before you a few desultory remarks on modern Pharmarcopœias, as a continuation of those admitted into the Medical and Physical Journal for April, which were chiefly applicable to the *Pharmacopoeia Edinburgensis*. These I now offer, although drawn from a different source, may be equally, if not more, acceptable to those whose opinion coincides with mine; I shall, therefore, without farther apology, submit them to your care.

In the preface to the "Edinburgh New Dispensatory," which may be considered as a very excellent translation of that from the college, it appears, that the honor, if there be any, of first adopting the new language in a dispensatory, belongs exclusively to the College of Edinburgh. This claim, I suspect, may be fairly disputed, were it worth the trouble; for if we look into *Pharmacopoeia Medici Practici Universalis*, lately published by Dr. Swediaur, we shall find, that the very last edition, with all the refinements of modern nomenclature, both of Chemistry and Materia Medica, have been adopted, in their most ample acceptation, by that author. If there be any difference it is, that as it has been employed much more extensively, there is, consequently, more confusion.

This Pharmacopœia is dated, "Paris, Feb. 16, 1803," which makes it prior to, if not coeval with, that of the Edinburgh College; it must, therefore, deserve, at least, an equal share of our acknowledgments for having adopted and cultivated this "pure language of science." This may be farther confirmed by attending to the *synonyma;* for when any thing is quoted from *Pharm. Edinb.* it is invariably from the *former* edition; thus *lixivia* is classed with the modern *potassa*. However, to compromise this matter, let us consider these works as contemporaries, and also that they form, as I shall venture to assume, two fair specimens of modern dispensatories, and of the vaunted perfection they have at last attained.

A very slight perusal of this Dispensatory will not permit me to wade through every page, and point out what I conceive to be either the whole of its defects or its perfections. As it is, however, of more serious importance to notice some of the former, lest silence should give currency to some glaring and very reprehensible errors, I

shall confine these cursory observations to one side of the question only with but little variation.

Through the whole work there appears an avowed intention, that a synoptical list, including all the *synonyma* of each composition, should always accompany and be placed immediately after the title. Had this scheme been properly accomplished and strictly followed, it must have proved a very useful, and in the present state of science, a most necessary guide.

In many instances we may observe, in this list, great confusion; in others, numberless mistakes; some names of the most interesting nature are entirely omitted; others of a most insignificant import carefully retained; and now and then we find no list whatever, where, perhaps, it was most required.

We may frequently observe the *synonyma* spun out with tedious minuteness, and a kind of drawling redundancy; including most of the obsolete and fantastic names; and what is unpardonable, omitting those of *modern* date. What has the present generation to do with the *alcahest de Van Helmont, arcanum corallinum, sal cardui benedicti,* or the *lana philosophica?* Would it not have added more to our knowledge to have left out such as these, and admit a *mercurius corrosivus sublimatus,* or a *hydrargyrus nitratus ruber?*

One would naturally conclude that in a work dedicated to the manes of four illustrious British physicians, as this is, the *synonyma* of at least all the *British* Pharmacopœias could not have escaped the author's notice; that our *creta preparata,* our *zincum calcinatum,* our *hydrargyrus muriatus,* and many other guiltless articles, might have deserved a situation. Had this been attended to, science might have received some support, and much more advantage, than can possibly accrue from the *cadmia fornacum,* a *pulvis principis,* and an endless farrago of absurdities, now generally expunged.

There is, I am sorry to say, one very serious case of the most reprehensible kind, that merits the attention of the whole medical world; whether the blame attach to this Pharmacopœia or to the Pharm. Edinb. is left to others to decide, and to deal out the *quantum meruit* to whom it may be due. I had scarcely opened the pages of Dr. Swediaur's work, when I perceived that the *murias hydrargyri* of the Edinburgh College was not the same as in this Pharmacopœia; that they were two very opposite things; in short, to speak plainly, that *murias hydrargyri* in one means means *corrosive sublimate of mercury;* and in the other, *calomel!!!*

This is surely no very trivial error, for the name of a mild and very manageable medicament in one country, to signify a direct poison in another! A blunder of this kind cannot be supposed to be corrected by an apothecary, whose business is to follow implicitly the prescription; but were it otherwise, as I am from the above instance persuaded it ought, *he* may be absent, and his inexperienced tyro may not always have so much faith in his own competency, to act beyond a certain sphere.

The *acetum distillatum* and *acidum acetosum,* both of the London Dispensatory, are admitted by this author as one and the same; the *acide acetique* of the French is likewise comprehended with the former. The *acidum aceticum concentratum* has but one solitary parallel or yoke-fellow, in *acetum radicale.* Here the author should have placed our *acid. acetos.* and the French *acide acetique.* What mischief may we not expect from such an arrangement of names! About four pages are dedicated to the acidum aceticum, and contain a jumble of about seven *formulæ,* in which it is difficult to guess whether a *distilled vinegar* or a *concentrated acetic acid* is to be produced; the last seems very obscure; *pure* vinegar to be distilled from manganese to form a *concentrated* acid.

Under the denomination of *oxyda metallica* are ranked the *scoriæ* metallorum; and, one would also suppose, the metallic *carbonates*; for with *oxydum ferri luteum* and *fuscum* the author classes both crocus martis *adstringens* and *aperiens.*

With *oxydum hydrarg. nigr.* our *hydrarg. cum creta* is placed, and *mercurius gummosus* is also added. Our *hydr. mur. mitis* is out of its place, and appears as if prepared by *sublimation.*

*Carbonas* and *oxydum zinci* are confounded both in this Pharmacopœia and that of the Edinburgh College; these are synonymized with very crude articles, such as *lapis tutiæ, lapis calamin. purificatus, &c.*

In this Pharmacopœia the word *stibium* is used throughout for *antimonium.* Many of the preparations from this metal are incorrectly and very carelessly detailed. *Tartris stibii* one would naturally conclude to be the exact parallel to *tartris antimonii* of *Pharm. Edinb.* but it is certainly not so, for the former is composed with *acid* of tartar only, and the latter with *cream* of tartar, or, agreeably to the ephemeral jargon, the super-tartrite of potash. So far the the author, in *his* name, has acted consistently. There is, however, another preparation admitted, which is analogous to that of the Edinburgh Dispensatory, the emetic tartar of former date, and is called *tartaris potassæ stibiatus;* though, to shew the versatility of the new diction, another preparation, in which oxyd of iron enters, and is in all respects analogous, is called *tartris ferri cum potassa.* This, it may be insisted on, implies tartrite of iron merely *mixed* with some potash, and not a *chemical union* of three things. The same observation may be applied to other similar *formulæ,* such as *oxydum stibii cum potassa,* for it is at best very ambiguous, since *cum* may signify either *with* or *by means of.*

The plan of the London College, though chosen perhaps inadvertently, seems better adopted to express a compound containing *cream* of tartar. Natron *tartarisatum,* antimon. *tartarisatum,* &c. might very well serve the present purpose, as *triple* compounds. As *binary* preparations, we might say *tartris,* or tartras (for this is also still indetermined) sodæ, or *tartris* antimonii, to shew that the *acid* of tartar only is employed.

Two methods are prescribed to make an oxyd of antimony (the *pulvis algorothi),* and detailed with as much appearance of precision, and under two distinct heads, as if there was no similitude between them, being separated by three or four other *formulæ.* One is *oxydum stibii album,* the other the same with *præcipitatum* added. In the first, it is one part of muriate of antimony to six or eight of distilled water; in the second there is a *quantum placet* to a *quantum opus.* These and many such proofs may be found in this dispensatory, that shew great carelessness and the slovenly want of attention that seems so generally to prevail. Indeed, a frequent occurrence of a quantum *satis, placet,* or *opus,* has but at best a very awkward appearance; and must always give one an idea of a closet-experiment.

By recent experiments it has been proved, that *animal bone,* that of quadrupeds in particular, contains *magnesia;* how far this may influence the nomenclature of our *pulvis antimonialis,* or of the improvement, the *phosphas calcis stibiatus,* I cannot determine. It is obvious, that not one of the modern Pharmacopœias contains a prescription for *pure* phosphate of lime; perhaps it may be unnecessary;—there has been, however, no attempt to try; and when it has been effected, we must be taught how it is to be *stibiated*

*ated* and become, as in *Ph. Edinb.* oxydum antimonii *cum* phosphate calcis.

Under the title *alcohola aromatisata*, the list of *synonyma* include what were formerly *spiritus stillatitii, aquæ spirituosæ, vinosæ*, and of course, the *spiritus anisi*, &c. of the London and Edinburgh dispensatories. Nothing can be more improper than this list; it had better have been omitted entirely; for it is worthy of remark, all the *alcohola* are to be made with *rectified* spirit; and all the *synonyma* are composed with *proof* spirit of wine. To complete this part of the work, *alcohol camphoratum* stands first in the list, though the formula is inserted elsewhere.

Amongst a list of *aquæ minerales* are the *aqua barytæ* and *aqua calcis*. I should seriously hope a poison of such energy as *barytic lime-water* may never be suffered to hold such a denomination as a *mineral* water in the general acceptation of this title. If a mineral water be made artificially, it is merely with a view to imitate *Nature;* and it may be boldly asserted, that nature never produced an aqueous solution either of lime or barytes. Moreover, mineral waters, if not drank always as a common beverage, are generally taken more in an *ad libitum* way than one would dare to give this *aqua barytæ*.

If acidum *citricum*, or *oxalicum*, be approved, we should by analogy adopt *tartaricum*, and not *tartarosum;*—the same observation may be applied to *citras* and *oxalas*, which may very fairly include *tartras* in place of *tartris*.

There is a *sulfis sodæ sulfuratus*, but no formula annexed to it: the source from which this is obtained proves it must be an extremely vague preparation, similar to *Bitnoben*, or to some such trash, and very unworthy a place in any dispensatory.

In more places than one, sulfurated hydrogen is rendered simply *gaz hydrogeneum*; and in the second process for *hydrosulfuretum ammoniæ* there seems a material omission: an acid is generally required to be added to sulfuret of potash, to disengage more effectually this gas. It is difficult to decide exactly the author's orthography of *sulphur*, whether he prefers it with *ph*, or an *f* only; perhaps I may have caught the contagion, and, confessing the effect it has, may also use both.

A *solution of potash in water* has nothing to distinguish it from one of the most potent caustics we possess. In this volume, *potassa* is followed by its supposed *synonyma*, in which are *aqua kali puri, aqu. lixiv. caustic.* and a few more of *liquid* alkali. In the present state of science,

*potassa*

*potassa* standing alone, must denote what the author does not mean to express, a *solid* simple substance. The danger might very easily have been obviated, potassa *aquosa,* or *soluta,* could not have lengthened it too much, in a work where the length of names have not been measured too sparingly.

From the second process to prepare *baryta,* one would imagine, by the "unde acidum nitricum fugatur," that nitric acid is not decomposed but evolved entire, and might be saved: this, however, is known to be otherwise; and that the acid is destroyed, the nitrogene and oxygene being extricated.

In *pilulæ hydrargyri,* I cannot discover whether each pill is to contain four grains of the *oxyd of mercury,* or four grains of the whole *mass:* if the former be meant, *fiant pilulæ quindecim* would have made it perspicuous; but if it be the *mass,* the prescription is very vague, and wants precision. I may here add, that unless *muriate, acetate, phosphate,* and the *tartrite* of mercury be equivalent to each other in strength, all that has been written respecting this pill the author should revise.

*Acidum sulphuricum* is accompanied by some instructions to purify it, which, if I mistake not, may render it very unfit for many purposes. The author says, "Si heterogeneis inquinatum sit, ex retorta vitrea vel *ferrea* destillari debet, &c." I should suppose sulphuric acid cannot be rendered pure if it be drawn from an *iron* retort; it must be highly contaminated by *sulphurous* acid; its specific gravity much lessened, and part of the acid lost in forming sulphate of iron, by abrading the retort.

There is a singular *nota bene* attached to the *formula* for acidum nitricum *concentratum.* In order to purify this acid, so very essential to many of the most important operations in chemistry, which require it not only strong but pure; the author directs the usual stale method, to drop in nitrate of silver to separate muriatic acid. This we know will produce the effect, but requires great address to leave no nitrate of silver in the acid, which, by the way, is never *strengthened* by this addition.

The next task to be performed is, to drop into the same acid a solution of *nitrate of barytes*; and this we are advised to continue, "*donec nihil amplius præcipitetur.*"— Now this I affirm to be a complete closet-experiment, or that the whole must have been penned and built upon theory: had the author been possessed of a few practical *præcognita,* he must have known better. Most of our systematic systematic books have retailed the same trite experiment; and, by trusting to some of these, our author has probably been deceived.

There never can exist any nitrous, or nitric acid, however pure, and even when perfectly freed from sulphuric acid, in which nitrate of barytes does not precipitate; and this precipitation will continue as long as we keep adding, till much more of the solution has been used than the quantity of acid it is meant to purify.

Besides this, would it not be highly absurd to add *concentratum* to an acid, into which so much *water* had been thrown?

That the *muriate* of barytes is also equally insoluble in *muriatic* acid, was, I have reason to believe, first discovered by myself: both this and the above fact respecting the nitrate, with some other peculiarities of barytes, have already been communicated to the public, through the medium of the Philosoph. Magazine.

Some modern Pharmacopœias have certainly been compiled under a most baneful influence of innovation, and a strong propensity to instability, veering rapidly from one point of nomenclature to another; neither knowing where to stop nor where to begin.

It must generally be acknowledged, that even a trifling change in any pharmaceutic composition, whether it be in the name, process, or the proportions of its constituent materials, must, for the most part, be attended with at least some inconvenience, if not danger. These must of course be commensurate with the importance of such compound: thus, when *mercury*, *opium*, or *antimony* forms the predominant article, we should be more scrupulous to admit any deviation, than where less significant ingredients are employed.

I do not wish to be understood, as having given a decision upon all cases of disparity that occur in modern Pharmacopœias; nor to be considered as opposing the new nomenclature *in toto*. That there is much confusion in the present language; that it is often perverted; often inconsistent with itself; full of ambiguities; very inadequate to the purposes of pharmacy; and evidently, from what has been said, perplexing to the pharmacopolist, are palpable truths, confirmed by daily experience.

Unwarrantable innovations and multiform titles are very incompatible with true science, and ought to be universally exposed and resisted. The length of some titles is preposterous beyond all bounds. Can we obtain an oil from from any other part of the *fruit* of the orange tree, that we must be constrained to say, *Oleum volatile corticis flavi citrus-aurantii?* Is there so much danger in retaining the *kermes mineralis* as to have recourse to *oxydum stibii hydrosulfuratum rubro-fuscum?*

I do not profess to be one of those who look forward to, and are constantly endeavouring to cultivate, a kind of finical perfection in nomenclature; my opposition arises from more pure motives. My humble opposition is founded on a strong desire to stem this torrent of novelty; to lessen a number of notorious and dangerous blunders; to discourage subsultory editions of national pharmacy, while the storm of innovation continues; and to advise those whom it most concerns, either to retrace a great deal, if not the whole, of what has lately been promulgated, or rest on their oars till they can proceed with more safety and more propriety. I am, &c.

J. HUME.

*Long Acre, June* 14, 1804.

---

